# Securing the U.S. nucleic acid synthesis industry: a case for safe-harbor information sharing

**DOI:** 10.3389/fbioe.2026.1819510

**Published:** 2026-05-15

**Authors:** Meghan J. Seltzer, Alice R. Isenberg, Ayanna Flegler, David W. Walburger, Matthew Eric Downs, John Dileo

**Affiliations:** The MITRE Corporation, McLean, VA, United States

**Keywords:** biosecurity, information sharing network, order screening, sequence of concern, synthetic nucleic acid

## Abstract

The U.S. has issued guidelines for synthetic nucleic acid providers to screen order sequences and customers. However, these guidelines do not provide clear, enforceable requirements for the industry and place significant responsibility on providers to appropriately interpret and apply these guidelines. This, in turn, can lead to uneven implementation, inconsistent risk assessments, and exploitable vulnerabilities. We propose that a safe harbor information sharing network could function as a biosecurity force multiplier. Such a system would enable cross-provider analysis of order sequences and customer metadata, improve detection of suspicious activity, generate empirical evidence to refine definitions of sequences of concern and screening priorities, and provide a more robust foundation for the iterative improvement of policies, guidelines, and regulations.

## 1 Introduction

Since the early 2000s, industry, academic, and government stakeholders have taken notable and distinct steps to secure the synthetic nucleic acid industry (Executive Office of the President of the United States, 2024; U.S. Department of Health and Human Services Administration for Strategic Preparedness and Response, 2023; International Gene Synthesis Consortium, 2020). However, current guidelines and frameworks are voluntary, and synthetic nucleic acid providers bear the responsibility to correctly interpret guidelines, assess risk, and identify threats. A comprehensive system to secure the synthetic nucleic acid marketplace must provide clear requirements and tools to conduct sequence screening and customer vetting; clear requirements for reporting potential suspicious behavior to appropriate law enforcement agencies; and processes to validate practices and tools.

In this article, we summarize the current synthetic nucleic acid order screening policies and highlight gaps that can lead to increased risk within the industry. Additionally, we propose that a safe harbor information sharing network could serve as a force-multiplier to further secure the synthetic nucleic acid industry by promoting information sharing and serving as a test bed for the development and refinement of policies and regulations.

## 2 Gaps in U.S. regulations, policies, and best practices

Currently, the U.S. Government has issued voluntary guidelines to help ensure the security of the synthetic nucleic acid industry. In 2010, the U.S. Department of Health and Human Services (HHS) issued guidance to synthetic nucleic acid providers to screen order sequences for sequences of concern (SOCs). This guidance was updated in 2023 and was supplemented by guidance from the White House Office of Science and Technology Policy (OSTP) in 2024 (U.S. Department of Health and Human Services Administration for Strategic Preparedness and Response, 2023; Executive Office of the President of the United States, 2024). In 2025, President Trump issued an executive order requiring that the 2024 Framework for Nucleic Acid Synthesis Screening be revised to further reduce the risk of misuse (Executive Office of the President of the United States, 2025b). At the writing of this article, however, updated guidance from the White House has yet to be issued.

In addition to U.S. guidance, the International Gene Synthesis Consortium (IGSC) has developed its Harmonized Screening Protocol (International Gene Synthesis Consortium, 2020), which provides guidance to its members on proper screening protocols. Two International Organization for Standardization (ISO) standards—ISO 20688-1 and ISO 20688-2—also describe basic requirements for providers to institute a sequence screening mechanism (International Organization for Standardization, 2020; International Organization for Standardization, 2024).

Existing guidelines and standards, however, have left notable gaps which can create confusion and lead to uneven implementation of processes, inconsistent risk assessment, and mitigation (Wheeler et al., 2024). These gaps include requirements to conduct order screening, clear definitions of what constitutes a sequence of concern (SOC), uniform procedures for vetting customers, triggers for reporting to law enforcement, reliance on vendor self-attestations, and a focus on actions of individual providers rather than the industry as a whole.

### 2.1 Lack of a requirement to screen

To date, there is no enforceable requirement for providers to follow the existing U.S. guidance. The 2024 OSTP Framework requires that organizations conducting federally funded research must use federal funds to purchase synthetic nucleic acids from providers that adhere to the framework (Executive Office of the President of the United States, 2024). Providers could, in theory, not follow this requirement. However, providers operating in the U.S. would be excluded from participation in a significant portion of the U.S. market if they did not supply orders to federally funded institutions.

Additionally, providers must also ensure that they are not providing certain synthetic nucleic acids to persons not authorized to receive them, regardless of funding source, in accordance with U.S. laws and regulations. These include regulated sequences (e.g., select toxins) and export-controlled sequences (Federal Select Agent Program, 2026; U.S. Congress, 2004; U.S. Department of Commerce, 2026; U.S. Department of State, 2026). Therefore, providers must, at a minimum, screen for these sequences to prevent violating U.S. regulations and laws.

### 2.2 Unclear definitions of sequences of concern

Current definitions of SOC are unclear and can lead to uneven assessment of their risk (Beal and Alexanian, 2025; Wheeler et al., 2024) as seen in Table 1. These definitions include vague statements that leave significant room for interpretation as to whether a sequence contributes to pathogenicity or toxicity; and expands to sequences that are not associated with infectious agents or biologically derived toxins. Additionally, current guidance often places the responsibility on the provider to make the determination of whether a sequence is an SOC or not. The lack of objective clarity can place providers at risk of violating regulations and guidelines and at risk of losing customers, if the risk assessment is not calibrated appropriately (Beal and Alexanian, 2025; Sequence Biosecurity Risk Consortium, n.d.).

**TABLE 1:** Comparison of sequence of concern definitions across several frameworks.

Source	Sequence of concern definition
2023 HHS screening framework guidance for providers and users of synthetic nucleic acids (U.S. Department of Health and Human Services Administration for Strategic Preparedness and Response, 2023)	A nucleotide sequence that is a best match … to a sequence of federally regulated agents (i.e., the Biological Select Agents and Toxins List, or the CCL), except when the sequence is also found in an unregulated organism or toxin. As soon as it is practical to do so, it is also recommended that sequences known to contribute to pathogenicity or toxicity, even when not derived from or encoding regulated biological agents, be treated as SOCs.
Framework for nucleic acid synthesis screening (Executive Office of the President of the United States, 2024)	Nucleotide sequence or its corresponding amino acid sequence that is a Best Match to a sequence of federally regulated agents (i.e., the Biological Select Agents and Toxins List (BSAT) or the Commerce Control List (CCL)), except when the sequence is also found in an unregulated organism or toxin. As of and after 13 October 2026, this definition will include sequences known to contribute to pathogenicity or toxicity, even when not derived from or encoding regulated biological agents.
	Additionally, as of 13 October 2026, this framework also requires providers to implement mechanisms to screen these additional SOCs, which include reviewing scientific evidence regarding pathogenicity or toxicity; the degree to which the sequence is likely to be recognized as a candidate for misuse, and the ease with which this sequence could be misused.
Harmonized screening protocol (International Gene Synthesis Consortium, 2020)	A broad term often used to capture the ideal not just of sequences currently subject to formal regulatory control but any sequence that can be misused to cause significant harm.

Both the IGSC and the Sequence Biosecurity Risk Consortium (SBRC) have undertaken efforts to provide guidance on SOCs to the community. The IGSC developed, and annually updates, a restricted pathogen database for use by its members (International Gene Synthesis Consortium, 2020). Additionally, the SBRC aims to maintain a “standard definition of ‘sequences of concern’ based on a scientific assessment of biosecurity risk” through development of a biosecurity flag rubric (e.g., no flag, flag, and undefined) and associated data test sets (Beal and Alexanian, 2025; Sequence Biosecurity Risk Consortium, n.d.).

### 2.3 Unclear customer vetting requirements

Similarly, these guidelines have limitations in determining both customer legitimacy (e.g., identity verification) and whether their described use is acceptable. 1mportantly, verification of customer legitimacy is only required when a customer orders an SOC. The OSTP Framework and the 2023 HHS Guidance specify that providers must verify that customers are legitimate and have a legitimate need through (Executive Office of the President of the United States, 2024; U.S. Department of Health and Human Services Administration for Strategic Preparedness and Response, 2023).

- Collecting and reviewing information such as name, institution, and contact information
- Collecting and reviewing information such as proposed use, institutional approval information, publication history, or regulatory permits
- Screening against the U.S. Consolidated Screening List (U.S. International Trade Administration, n.d.) (e.g., restricted party screening)
- Ensuring that orders do not raise red flags such as unclear or fraudulent customer identity; mismatch between proposed use and job responsibilities and institutional affiliation; unusual labeling, shipping, or payment requests; or unusual requests for confidentiality or order changes.

These guidelines note that providers must develop their own criteria for determining whether to fill an order based on the results of sequence and customer screening. As such, this process is highly dependent on the provider and their risk tolerance, which can lead to inconsistent risk assessment (International Gene Synthesis Consortium, 2020; Alexanian and Nevo, 2024). In life sciences research, DNA sequences are often ordered by one person on behalf of a broader research laboratory or collaborative group; so the provider that vets the buyer may not be screening all end users of the material. To address this problem, the HHS guidance requires customers to conduct due diligence on end users as well (U.S. Department of Health and Human Services Administration for Strategic Preparedness and Response, 2023).

There are a variety of open-source, free, and commercial tools exist to support providers for sequence screening (Alexanian and Nevo, 2024). In contrast, few resources exist to help support companies in verifying customer legitimacy and legitimate use. U.S. Government databases and commercial solutions exist to support restricted party screening (U.S. International Trade Administration, n.d.). However, there is a lack of similar tools to support determination of customer legitimacy.

Using the available guidelines, the International Biosecurity and Biosafety Initiative for Science has developed decision tools with flowcharts and decision trees to help providers work through the criteria to reach a finding (International Biosecurity and Biosafety Initiative for Science, n.d.). However, as these are based on existing guidelines, they do not help to address the inherent ambiguities.

### 2.4 Lack of a requirement to report suspicious behavior to law enforcement

Current U.S. guidelines encourage, but do not require, providers to report suspicious behaviors or suspicious orders to the Federal Bureau of Investigation Weapons of Mass Destruction Coordinators (Executive Office of the President of the United States, 2024; U.S. Department of Health and Human Services Administration for Strategic Preparedness and Response, 2023). Providers are required to report potential export control violations to the U.S. Department of Commerce Bureau of Industry and Security. However, there is generally no clear definition of suspicious behavior or orders, so providers develop or employ varying definitions (Engineering Biology Research Consortium, 2025). The OSTP Framework notes that if follow-up screening does not resolve sequence or customer screening concerns, providers should decline the order and report it to the appropriate authorities (Executive Office of the President of the United States, 2024).

### 2.5 Lack of order screening certification processes

Current U.S. policy requires providers to self-certify that they adhere to policies, which only offers limited assurance to regulators, customers, and the industry writ large (Executive Office of the President of the United States, 2024). For example, the OSTP Framework notes that providers must note on their website or provide certification to federally funded customers that they comply with the framework (Executive Office of the President of the United States, 2024). However, the framework offers little guidance on what constitutes adherence or compliance, especially given the ambiguities discussed above. Additionally, the HHS Framework places the onus on the provider to periodically test and measure the effectiveness of their processes, again providing little guidance how to accomplish this goal (U.S. Department of Health and Human Services Administration for Strategic Preparedness and Response, 2023; U.S. Department of Health and Human Services, 2023; Engineering Biology Research Consortium, 2025).

Therefore, in the absence of defined standards and metrics to certify compliance with existing standards, providers are responsible for assessing and determining whether they comply with the standards. This can result in uneven implementation, weakened accountability, and increased reputational risk for providers. Development of a certification process and identification of a certifying body could help providers align practices and increase consistency in practices between providers.

### 2.6 Focus on individual provider actions and individual orders

In addition to the lack of clarity, existing guidelines focus on individual providers screening individual orders. This provider-centric model increases the risk for “split-order attacks” where customers divide orders across multiple providers over time to conceal SOCs or their identity. Additionally, monitoring activity at the individual order or provider level would not detect certain types of suspicious activity such as “venue shopping” where individuals attempt to order sequences from multiple or alternative providers when their orders are declined or questioned.

## 3 Safe harbor information sharing network as a biosecurity force multiplier

A safe harbor information sharing network for the nucleic acid synthesis industry could help address the key gaps. Safe harbor networks include provisions for participants to share critical information while protect participants from liability or penalties in accordance with agreements, regulations, or statutes without fear of repercussions (Cornell Law School Legal Information Institute, n.d.). The Aviation Sharing Information Analysis and Sharing (ASIAS) network is a well-known example of a safe harbor information sharing system through which aviation companies share safety data to enable detection of system-wide aviation hazards (The MITRE Corporation, 2025).

Interest in establishing a safe harbor information sharing network for the nucleic acid synthesis industry and the bioeconomy more broadly has gained considerable interest in the past few years (Beal and Alexanian, 2025; National Security Commission on Emerging Biotechnology, 2025; Watson et al., 2024; Office of Senator Tom Cotton, 2026). Notably, America’s AI Action Plan, released in 2025, recommends that OSTP “convene government and industry actors to develop a mechanism to facilitate data sharing between nucleic acid synthesis providers to screen for potentially fraudulent or malicious customers” (Executive Office of the President of the United States, 2025a).

Existing consortium-based models have enabled standards development and sharing of best practices. However, consortia often do not have systems for providers to share critical information required to identify split-order attacks or suspicious behavior patterns. This information could include proprietary customer or company information (e.g., proprietary sequences), customer metadata, or descriptions of possible illegal activity. Customer and provider acceptance of information sharing is more likely if the network collects the minimum amount of data needed to achieve its purpose, employs privacy-preserving protections supported strong data use agreements and cybersecurity controls, and has a strong governance structure—features that are standard in safe harbor networks.

Establishing a safe harbor information sharing network could strengthen biosecurity by analyzing data across providers to detect potential split-order attacks or suspicious behavior; enhancing understanding and characterizing risks associated with the industry; and providing insights to inform development of policies, guidelines, regulations, and best practices (Beal and Alexanian, 2025; Watson et al., 2024). For example, providers sharing order information (e.g., sequence information and customer metadata) with the network could lead to data analysis in real time to identify potential split-order attacks or suspicious behavior. This analysis could also provide useful information to the industry on the estimated frequency of such events and help define the most common threat vectors from nefarious actors. The network could generate reports and issue alerts to inform provider practices, allowing alignment of efforts and scrutiny with better defined risk profiles.

Additionally, the same information could be used to better inform efforts to identify or define potential SOCs. Historical data across providers can reveal how often specific sequences are ordered and highlight sequences that are commonly ordered but have inconsistent classification as an SOC among providers. As organizations such as the SBRC and IGSC work to define SOCs or update SOC lists, this historical information could help identify or prioritize sequences that require additional guidance or a formal determination as to whether the sequence should be listed as an SOC.

Developing a safe harbor information sharing network will require careful design to ensure that this network adds overall benefit to the system without exposing providers to additional liability, antitrust risks, or a competitive disadvantage. Strong data protection and cybersecurity measures will be essential to comply with data sharing laws and restrictions and protect shared information.

Additionally, for the network to achieve its goals, provider participation must be high and participation should benefit individual companies. The value proposition for participation will vary depending on whether the system is mandated by regulation or policy or voluntary. For example, in a mandatory paradigm, companies would be incentivized to participate to avoid civil or criminal penalties or to avoid loss of certain types of business (e.g., federally funded research). In a voluntary paradigm, strong incentives and benefits would need to be developed to encourage participation.

## 4 Discussion

The current U.S. biosecurity framework for the synthetic nucleic acid industry is a patchwork of voluntary guidelines that establish basic expectations for synthetic nucleic acid order screening but lack clear requirements and guidance for several key aspects. The current system places heavy responsibility on providers, leading to varied practices, inconsistent risk assessments, and exploitable gaps. These weaknesses are compounded by a narrow focus on provider- and order-centric processes. Malicious actors can potentially evade safeguards through tactics like split-order attacks, venue shopping between providers with different standards, or exploiting differences in risk tolerance and screening tools.

The U.S. Government is currently undertaking efforts to improve these gaps. Such efforts include the revision of the OSTP Framework under the Improving the Safety and Security of Biological Research Executive Order (Executive Office of the President of the United States, 2025b), biosecurity improvements noted in America’s AI Action Plan (Executive Office of the President of the United States, 2025a), and the introduction of The Biosecurity Modernization and Innovation Act in the U.S. Senate (Office of Senator Tom Cotton, 2026). If passed, this Act would improve adherence to screening frameworks by requiring the Department of Commerce to develop and maintain a list of SOCs, develop conformity assessment systems to verify provider adherence to the regulations, and establish regulations requiring providers to implement screening protocols for SOCs, including cross-provider order screening to detect split orders (Office of Senator Tom Cotton, 2026).

While this article focuses on the current U.S. landscape, it is important to note that U.S.-based providers that sell products internationally must also comply with international guidance and requirements. These frameworks include country-specific or regional requirements for nucleic acid screening (e.g., UK screening guidance on synthetic nucleic acids for uses and providers or EU Biotech Act) and a patchwork of export and import requirements for both synthetic nucleic acids and their associated data. As a result, providers must navigate increasingly complex requirements depending on their location, their customer’s location, and funding used for the purchase.

Establishing a safe harbor information sharing network for the synthetic nucleic acid synthesis industry would help close potential security gaps and develop an evidence base to inform existing policies. A safe harbor information sharing network could enable analysis of sequences and customer metadata to detect suspicious patterns, measure the frequency and nature of threats, and generate evidence to refine definitions of sequences of concern and screening priorities. In parallel, an independent certification process—supported by elements such as standardized test sets, performance metrics, proficiency testing, and independent evaluation of provider practices—could help align practices and increase consistency between providers. Over time, this network would provide a wealth of data and insights to help inform further development and refinement of guidelines to more effectively secure the synthetic nucleic acid synthesis industry.

## Data Availability

The original contributions presented in the study are included in the article/supplementary material, further inquiries can be directed to the corresponding author.

## References

[B1] Alexanian T. Nevo S. (2024). Supporting follow-up screening for flagged nucleic acid synthesis orders. Available online at: https://councilonstrategicrisks.org/2024/05/07/supporting-follow-up-screening-for-flagged-nucleic-acid-synthesis-orders/(Accessed Feburary 27, 2026).

[B2] Beal J. Alexanian T. (2025). Creating enforceable biosecurity standards for nucleic acid providers. Eng. Biol. 9 (1), e70003. 10.1049/enb2.70003 41415623 PMC12710665

[B23] Cornell Law School Legal Information Institute (n.d,). Safe Harbor. Available online at: https://www.law.cornell.edu/wex/safe_harbor (Accessed March 23, 2026).

[B3] Engineering Biology Research Consortium (2025). Strengthening a safe and secure nucleic acid synthesis ecosystem: outcomes of ebrc stakeholder engagement. Engineering Biology Research Consortium. 10.25498/E4311B

[B4] Executive Office of the President of the United States (2024). Framework for Nucleic Acid Synthesis Screening. United States: OSTP.

[B5] Executive Office of the President of the United States (2025a). America’s AI Action Plan.

[B6] Executive Office of the President of the United States (2025b). Improving the safety and security of biological research. Available online at: https://www.whitehouse.gov/presidential-actions/2025/05/improving-the-safety-and-security-of-biological-research/(Accessed Feburary 27, 2026).

[B7] Federal Select Agent Program (2026). Federal select agent program. Available online at: https://www.selectagents.gov (Accessed Feburary 27, 2026).

[B8] International Biosecurity and Biosafety Initiative for Science (IBBIS) (n.d.). Customer screening. Available online at: https://ibbis.bio/our-work/customer-screening/(Accessed Feburary 27, 2026).

[B9] International Gene Synthesis Consortium (2020). International gene synthesis consortium. Available online at: https://genesynthesisconsortium.org/(Accessed Feburary 27, 2026).

[B10] International Organization for Standardization (2020). ISO 20588-1:2020 Biotechnology - Nucleic Acid Synthesis Part 1: Requirements for the Production and Quality Control of Synthesized Oligonucleotides. Geneva, Switzerland: International Organization for Standardization in Vernier.

[B11] International Organization for Standardization (2024). ISO 20688-2:2024 Biotechnology - Nucleic Acid Synthesis Part 2 Requirements for the Production and Quality Control of Synthesized Gene Fragments, Genes, and Genomes. Geneva, Switzerland: International Organization for Standardization in Vernier.

[B12] National Security Commission on Emerging Biotechnology (2025). Charting the Future of Biotechnology: an Action Plan for American Security and Prosperity.

[B13] Office of Senator Tom Cotton (2026). Cotton, Klobuchar introduct bill to establish federal Biotech security framework. Available online at: https://www.cotton.senate.gov/news/press-releases/cotton-klobuchar-introduce-bill-to-establish-federal-biotech-security-framework (Accessed Feburary 27, 2026).

[B14] Sequence Biosecurity Risk Consortium (n.d.). Sequence biosecurity risk consortium. Available online at: sbrc.bio (Accessed Feburary 27, 2026).

[B24] The MITRE Corporation (2025). Aviation safety information analysis and sharing. Available online at: https://www.asias.aero (Accessed March 23, 2026).

[B15] U.S. Congress (2004). 18 USC 175c: Variola Virus.

[B16] U.S. Department of Commerce (2026). 15 CFR parts 730-774: Export Administration Regulations.

[B17] U.S. Department of Health and Human Services (2023). Companion Guide to Assist in Implementing the Recommendations of the Screening Framework Guidance for Providers and Users of Synthetic Nucleic Acids.

[B18] U.S. Department of Health and Human Services Administration for Strategic Preparedness and Response (2023). Screening Framework Guidance for Providers and Users of Synthetic Nucleic Acids.

[B19] U.S. Department of State (2026). 22 CFR parts 120-130: International Traffic in Arms Regulations.

[B20] U.S. International Trade Administration (n.d.). Consolidated screening list. Available online at: https://www.trade.gov/consolidated-screening-list (Accessed Feburary 27, 2026).

[B21] Watson M. Rambhia K. J. Seltzer M. J. Carter S. R. Moritz R. L. Attal-Juncqua A. (2024). Toward a safer and more secure US bioeconomy. Nature Biotechnol. 43, 23–25. 10.1038/s41587-024-02519-2 39681703

[B22] Wheeler N. Bartling C. Carter S. R. Clore A. Diggans J. Flyangolts K. (2024). Progress and prospects for a nucleic acid screening Test set. Applied Biosafety 29 (3), 133–141. 10.1089/apb.2023.0033 39372513 PMC11447130

